# Individual and joint influence of cytokeratin 19 and microvascular invasion on the prognosis of patients with hepatocellular carcinoma after hepatectomy

**DOI:** 10.1186/s12957-022-02632-z

**Published:** 2022-06-21

**Authors:** Shang-Dong Qin, Jie Zhang, Ya-Peng Qi, Jian-Hong Zhong, Bang-De Xiang

**Affiliations:** 1 Hepatobiliary Surgery Department, Guangxi Medical University Cancer Hospital, Nanning, Guangxi China; 2 Key Laboratory of Early Prevention and Treatment for Regional High Frequency Tumor (Guangxi Medical University), Ministry of Education; Guangxi Key Laboratory of Early Prevention and Treatment for Regional High Frequency Tumor, Nanning, Guangxi China; 3 Affiliated Cancer Hospital of Zhengzhou University, Zhengzhou, China

**Keywords:** Hepatocellular carcinoma, Cytokeratin 19, Microvascular invasion, Radical resection

## Abstract

**Background and objectives:**

To evaluate the individual and combined associations of cytokeratin 19 (CK19) and microvascular invasion (MVI) with prognosis of patients with hepatocellular carcinoma (HCC).

**Methods:**

Clinicopathological data on 352 patients with HCC who underwent radical resection at our hospital between January 2013 and December 2015 were retrospectively analyzed. Patients were divided into four groups: CK19(−)/MVI(−), CK19(−)/MVI(+), CK19(+)/MVI(−), and CK19(+)/MVI(+).

**Results:**

Of the 352 HCC patients, 154 (43.8%) were CK19(−)/MVI(−); 116 (33.0%), CK19(−)/MVI(+); 31 (8.8%), CK19(+)/MVI(−); and 51 (14.5%), CK19(+)/MVI(+). The disease-free survival of CK19(−)/MVI(−) patients was significantly higher than that of CK19(−)/MVI(+) patients and CK19(+)/MVI(+) patients. Similar results were observed for overall survival. CK19(+)/MVI(+) patients showed significantly lower overall survival than the other three groups.

**Conclusions:**

CK19 expression and MVI predict poor prognosis after radical resection of HCC, and the two markers jointly contribute to poor OS. Combining CK19 and MVI may predict post-resection prognosis better than using either factor on its own.

Hepatocellular carcinoma (HCC) is one of the most common malignancies. Surgical resection is one of the most effective treatments for HCC [1]. However, the high rate of postoperative recurrence seriously affects prognosis [2]. For intermediate and advanced-stage HCC, the 5-year recurrence rate is up to 74% after hepatic resection [3]. The 5-year overall survival rate after hepatic resection is only 30% for those with intermediate disease and only 18% for those with advanced disease [4]. Official guidelines offer few adjuvant therapies to prevent HCC recurrence [5]. Key measures to improve prognosis may be stratification of HCC according to risk factors and effective intervention for patients with those factors. Therefore, it is important to study the risk factors that affect prognosis.

Microvascular invasion (MVI) is defined as the presence of cancer cell nests in the vascular cavity lined by endothelial cells under a microscope, including veins, arteries, and lymphatic vessels [6]. MVI is a marker of aggressive tumor behavior and is considered to be an important risk factor affecting the prognosis of patients with HCC [2, 6–12]. MVI significantly reduces disease-free survival (DFS) and overall survival (OS) of HCC patients, even after liver resection or transplantation [6].

Biliary cell markers including cytokeratin 19 (CK19) are also associated with poor prognosis after liver resection in HCC [13–19]. Similarly, CK19 predicts poor prognosis in HCC patients after liver transplantation [20, 21]. The association between CK19 and poor prognosis in HCC may reflect that the protein’s expression is closely related to lymphatic metastasis, which can lead to poor prognosis [18, 22], and to increased risk of portal vein invasion and bile duct cancer thrombosis [17, 23]. The OS of patients with CK19(+) HCC is similar to that of patients with combined HCC and cholangiocarcinoma (cHCC-CC) and higher than that of patients with intrahepatic cholangiocarcinoma (ICC), but lower than that of patients with CK19(−) HCC [24].

Given the association of both CK19 and MVI with poor prognosis in HCC, and given that combinations of biomarkers often predict outcomes better than single biomarkers on their own, we examined whether the two factors may help identify HCC patients at high risk of recurrence or death after hepatic radical resection.

## Patients and methods

### Patient information

This retrospective study involved patients with HCC who underwent radical resection at Guangxi Medical University Cancer Hospital between January 2013 and December 2015. The study protocol was approved by the Ethics Commission of Guangxi Medical University Cancer Hospital, which waived the requirement for informed consent because at the time of their surgery, all patients had consented for their anonymized medical records to be analyzed and published for research purposes.

To be included in the study, patients (1) had to be diagnosed with HCC that was confirmed by postoperative pathology; (2) had to be in Barcelona Clinic Liver Cancer (BCLC) stage 0, A or B; (3) had to have undergone radical resection; and (4) had to have complete follow-up information available. Radical resection of liver cancer was defined as surgery conducted without gross tumor thrombus in large vessels such as the hepatic or portal vein; without invasion of nearby organs, hilar lymph nodes or distant metastasis; with a resection margin lying more than 1 cm from the tumor boundary, or a resection margin ≤ 1 cm but without residual tumor cells at the margin; and with no detection of tumors by ultrasonography, computed tomography or magnetic resonance imaging at 1–2 months after surgery.

Patients were excluded if they had received other antitumor treatments before surgery, had a history of other tumors, or did not have complete pathology data available.

### Clinicopathological data

The following clinicopathological data were collected: age, sex, Barcelona Clinic liver cancer stage (BCLC stage), tumor diameter, tumor number, tumor envelope, ascites, hepatitis B surface antigen (HBsAg), hepatitis B virus DNA (HBV-DNA), antibodies against hepatitis C virus (Anti-HCV), white blood cell (WBC) count, hemoglobin (HB) level, neutrophil percentage (N%), lymphocyte percentage (L%), blood platelet (PLT) count, alpha fetoprotein (AFP) level, prothrombin time (PT), international normalized ratio (INR), fasting plasma glucose (FPG), total bilirubin (TBiL), albumin (Alb), Prealbumin (PA), alanine aminotransferase (ALT), aspartate aminotransferase (AST), γ-glutamyl transpeptadase (GGT), alkaline phosphatase (ALP), CK19 expression status, and MVI presence or absence.

CK19 status was determined by immunohistochemistry. CK19 positivity was defined as membranous and/or cytoplasmic expression in ≥ 5% of tumor cells with moderate or strong intensity. MVI status was determined by histopathology. MVI was defined as the presence of cancer cell nests in the vascular cavity lined by endothelial cells under a microscope. CK19 and MVI findings were retrieved retrospectively from pathological reports.

Enrolled patients were divided into four groups based on expression of CK19 and on the presence of MVI: CK19 (−)/MVI (−), CK19 (−)/MVI (+), CK19 (+)/MVI (−), and CK19 (+)/MVI (+).

### Follow-up

All patients were followed up until December 2019 or death. Tumor recurrence was diagnosed based on at least two imaging methods [25]. DFS was defined as the interval between the date of surgery and the date of diagnosis of tumor recurrence. OS was defined as the interval between the date of surgery and the date of death.

### Statistical analysis

Statistical analysis was performed using SPSS 23.0 (IBM, Chicago, IL, USA). Differences in categorical variables were assessed for significance using the chi-squared test. Differences in continuous variables were assessed using the *t* test, ANOVA, Mann-Whitney *U* test or Kruskal-Wallis *H* test, as appropriate, after determining whether data were normally distributed using the Shapiro-Wilk test and Q-Q plots.

DFS and OS were calculated using the Kaplan-Meier method, and differences in survival rate were assessed for significance using the log-rank test. Univariable analysis was conducted to identify factors significantly associated with DFS and OS, and variables that emerged as significant were entered in multivariable Cox proportional hazard modeling with forward stepwise selection. Differences associated with *P* < 0.05 were considered significant.

## Results

### Clinicopathological features of the study population

**Fig. 1:**
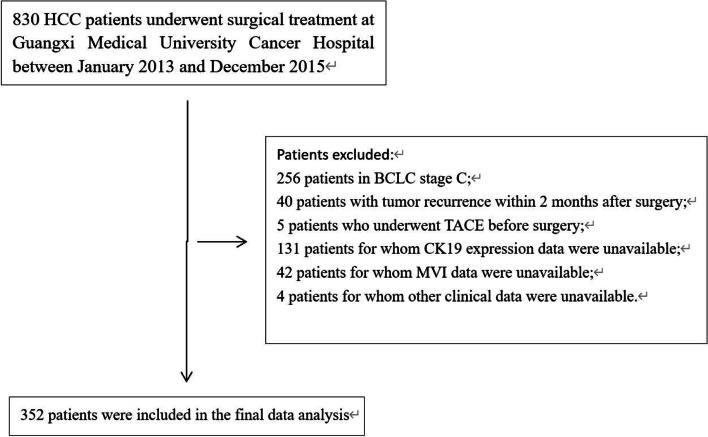
Enrollment and inclusion of patients. BCLC, Barcelona Clinic liver cancer; CK19, cytokeratin 19; HCC, hepatocellular carcinoma; MVI, microvascular invasion; TACE, transcatheter arterial chemoembolization

From 2013 to 2015, 830 patients with HCC underwent surgery at our hospital, of whom 352 were finally included in the analysis (Fig. 1). The median follow-up period was 51 months, during which 54 (15.3%) patients were lost to follow-up and 95 (27.0%) died. The average age was 49.2 years, and 83.5% of patients were male. CK19 expression was detected in 23.3% (82/352) patients; MVI was detected in 47.4% (167/352). The distribution of patients across the four groups was as follows: CK19(−)/MVI(−), 154 of 352 (43.8%); CK19(−)/MVI(+), 116 (33.0%); CK19(+)/MVI(−), 31 (8.8%); and CK19(+)/MVI(+), 51 (14.5%). The clinicopathological parameters of each group are described in Table 1.

**Table 1:** Associations of CK19 expression and presence of MVI with clinicopathological characteristics in patients with HCC

Characteristic	CK19(−)	CK19(+)	*P*	MVI(−)	MVI(+)	*P*	CK19(−)/MVI(−)	CK19(−)/MVI(+)	CK19(+)/MVI(−)	CK19(+)/MVI(+)	*P*
Age (years)	49.94 ± 10.51	46.76 ± 11.64	0.020	49.59 ± 10.80	48.75 ± 10.91	0.469	50.18 ± 10.48	49.62 ± 10.57	46.71 ± 12.04	46.78 ± 11.52	0.134
Sex											
Male	227 (84.1%)	67 (81.7%)	0.613	151 (81.6%)	143 (85.6%)	0.319	126 (81.8%)	101 (87.1%)	25 (80.6%)	42 (82.4%)	0.654
Female	43 (15.9%)	15 (18.3%)		34 (18.4%)	24 (14.4%)		28 (18.2%)	15 (12.9%)	6 (19.4%)	9 (17.6%)	
BCLC stage											
0	10 (3.7%)	5 (6.1%)	0.238	12 (6.5%)	3 (1.8%)	0.086	7 (4.5%)	3 (2.6%)	5 (16.1%)	0 (0%)	0.003
A	181 (67.0%)	60 (73.2%)		122 (65.9%)	119 (71.3%)		105 (68.2%)	76 (65.5%)	17 (54.8%)	43 (84.3%)	
B	79 (29.3%)	17 (20.7%)		51 (27.6%)	45 (26.9%)		42 (27.3%)	37 (31.9%)	9 (29.0%)	8 (15.7%)	
HBsAg											
Negative	35 (13.0%)	11 (13.4%)	1.000	20 (10.8%)	26 (15.6%)	0.207	16 (10.4%)	19 (16.4%)	4 (12.9%)	7 (13.7%)	0.549
Positive	235 (87.0%)	71 (86.6%)		165 (89.2%)	141 (84.4%)		138 (89.6%)	97 (83.6%)	27 (87.1%)	44 (86.3%)	
HBV-DNA (IU/ml)											
< 5 × 10^2^	90 (33.3%)	32 (39.0%)	0.356	63 (34.1%)	59 (35.3%)	0.823	51 (33.1%)	39 (33.6%)	12 (38.7%)	20 (39.2%)	0.823
≧5 × 10^2^	180 (66.7%)	50 (61.0%)		122 (65.9%)	108 (64.7)		103 (66.9%)	77 (66.4%)	19 (61.3%)	31 (60.8%)	
Anti-HCV											
Negative	266 (98.5%)	80 (97.6%)	0.627	182 (98.4%)	164 (98.2%)	1.000	152 (98.7%)	114 (98.3%)	30 (96.8%)	50 (98.0%)	0.896
Positive	4 (1.5%)	2 (2.4%)		3 (1.6%)	3 (1.8%)		2 (1.3%)	2 (1.7%)	1 (3.2%)	1 (2.0%)	
Tumor diameter (cm)	5.00 (3.50, 7.50)	4.75 (3.5, 8.00)	0.383	4.50 (3.50, 7.00)	5.50 (3.50, 8.50)	0.008	4.75 (3.50, 7.00)	6.00 (3.50, 8.50)	4.00 (2.5, 6.00)	5.00 (3.50, 10.00)	0.017
Tumor number											
1	193 (71.5%)	65 (79.3%)	0.200	140 (75.7%)	118 (70.7%)	0.335	117 (76.0%)	76 (65.5%)	23 (74.2%)	42 (82.4%)	0.098
≧2	77 (28.5%)	17 (20.7%)		45 (24.3%)	49 (29.3%)		37 (24.0%)	40 (34.5%)	8 (25.8%)	9 (17.6%)	
Tumor envelope											
Complete	230 (85.2%)	66 (80.5%)	0.305	164 (88.6%)	132 (79.0%)	0.019	134 (87.0%)	96 (82.8%)	30 (96.8%)	36 (70.6%)	0.008
Incomplete	40 (14.8%)	16 (19.5%)		21 (11.4%)	35 (21.0%)		20 (13.0%)	20 (17.2%)	1 (3.2%)	15 (29.4%)	
Ascites											
No	249 (92.2%)	73 (89.0%)	0.370	170 (91.9%)	152 (91.0%)	0.849	143 (92.9%)	106 (91.4%)	27 (87.1%)	46 (90.2%)	0.742
Yes	21 (7.8%)	9 (11.0%)		15 (8.1%)	15 (9.0%)		11 (7.1%)	10 (8.6%)	4 (12.9%)	5 (9.8%)	
AFP (ng/ml)											
<400	180 (66.7%)	31 (37.8%)	< 0.001	129 (69.7%)	82 (49.1%)	< 0.001	115 (74.7%)	65 (56.0%)	14 (45.2%)	17 (33.3%)	< 0.001
≥ 400	90 (33.3%)	51 (62.2%)		56 (30.3%)	85 (50.9%)		39 (25.3%)	51 (44.0%)	17 (554.8%)	34 (66.7%)	
WBC (*10^9^/L)	6.06 (4.88, 7.22)	5.40 (4.55, 6.80)	0.050	5.73 (4.66, 7.12)	6.06 (4.91, 7.16)	0.246	5.94 (4.74, 7.26)	6.17 (5.13, 7.16)	5.18 (4.55, 6.48)	5.73 (4.53, 7.31)	0.094
N% (%)	57.91 ± 9.96	57.17 ± 9.96	0.557	56.95 ± 9.87	58.61 ± 10.01	0.118	57.21 ± 9.96	58.83 ± 9.93	55.45 ± 9.42	58.09 ± 10.26	0.352
L% (%)	29.93 ± 8.47	30.05 ± 9.06	0.910	30.24 ± 8.72	29.64 ± 8.48	0.510	30.09 ± 8.49	29.72 ± 8.47	31.03 ± 9.87	29.45 ± 8.57	0.855
HB (g/L)	137.02 ± 17.20	140.01 ± 17.43	0.170	135.28 ± 17.43	140.43 ± 16.74	0.005	134.30 ± 17.08	140.64 ± 16.75	140.13 ± 18.63	139.94 ± 16.85	0.012
PLT (*10^9^/L)	209.92 ± 77.74	198.43 ± 81.61	0.247	208.52 ± 83.84	205.82 ± 72.79	0.749	211.92 ± 85.27	207.25 ± 66.72	191.61 ± 75.35	202.57 ± 85.66	0.585
FPG (mmol/L)	4.71 (4.31, 5.22)	4.73 (4.28, 5.14)	0.792	4.76 (4.31, 5.22)	4.66 (4.30, 5.16)	0.326	4.74 (4.29, 5.22)	4.68 (4.33, 5.22)	4.84 (4.40, 5.24)	4.65 (4.23, 5.10)	0.605
PT (s)	13.09 ± 1.11	13.31 ± 1.25	0.131	13.19 ± 1.11	13.09 ± 1.19	0.435	13.17 ± 1.07	12.99 ± 1.15	13.29 ± 1.30	13.32 ± 1.24	0.280
INR	1.06 ± 0.12	1.07 ± 0.13	0.441	1.06 ± 0.12	1.06 ± 0.12	0.804	1.06 ± 0.12	1.06 ± 0.12	1.08 ± 0.14	1.07 ± 0.12	0.838
TBiL (μmol/L)	11.40 (8.38, 15.53)	11.30 (8.10, 14.83)	0.549	11.00 (8.10, 15.30)	11.60 (8.70, 15.30)	0.402	10.90 (8.10, 15.23)	11.75 (9.40, 16.58)	13.20 (8.10, 16.20)	11.20 (8.10, 13.80)	0.359
Alb (g/L)	41.08 ± 4.28	42.12 ± 4.45	0.056	41.16 ± 4.01	41.50 ± 4.48	0.453	40.91 ± 3.87	41.31 ± 4.78	42.40 ± 4.49	41.95 ± 4.47	0.220
PA (mg/L)	197.50 (156.75, 244.25)	192.00 (139.00, 247.00)	0.610	203.00 (157.00, 243.00)	191.00 (150.00, 249.00)	0.691	198.50 (157.50, 242.00)	195.50 (154.75, 255.25)	218.00 (139.00, 257.00)	180.00 (139.00, 233.00 )	0.555
ALT (U/L)	34.50 (24.00, 47.00)	30.50 (22.00, 39.00)	0.099	33.00 (22.00, 45.50)	33.00 (24.00, 44.00)	0.936	32.50 (22.00, 46.00)	36.50 (24.25, 47.75)	34.00 (22.00, 41.00)	30.00 (22.00, 39.00)	0.196
AST (U/L)	36.00 (27.00, 49.00)	33.00 (25.50, 45.25)	0.195	33.00 (25.50, 46.00)	36.00 (29.00, 52.00)	0.032	35.00 (26.00, 47.00)	37.00 (29.00, 53.75)	29.00 (23.00, 40.00)	35.00 (28.00, 48.00)	0.047
GGT (U/L)	53.00 (33.00, 104.00)	54.00 (31.75, 91.75)	0.692	50.00 (29.00, 92.00)	57.00 (36.00, 105.00)	0.026	51.00 (30.00, 96.50)	57.00 (36.00, 109.00)	43.00 (26.00, 74.00)	57.00 (36.00, 104.00)	0.125
ALP (U/L)	63.00 (50.00, 81.00)	61.00 (45.00, 84.25)	0.638	59.00 (45.00, 78.50)	66.00 (53.00, 88.00)	0.016	59.50 (47.00, 78.00)	67.00 (55.25, 92.75)	56.00 (42.00, 85.00)	64.00 (48.00, 84.00)	0.085
MVI											
Negative	154 (57.0%)	31 (37.8%)	0.002								
Positive	116 (43%)	51 (62.2%)									

Values are mean ± SD, *n* (%), or median (interquartile range), unless otherwise noted

*Abbreviations*: *AFP* alpha-fetoprotein, *Alb* albumin, *ALP* alkaline phosphatase, *ALT* alanine aminotransferase, *anti-HCV* antibodies against hepatitis C virus, *AST* aspartate aminotransferase, *BCLC* Barcelona Clinic liver cancer, *CK19* cytokeratin 19, *FPG* fasting plasma glucose, *GGT* γ-glutamyl transpeptidase, *HB* hemoglobin, *HBsAg* hepatitis B surface antigen, *INR* international normalized ratio, *L%* lymphocyte percentage; *N%* neutrophil percentage, *MVI* microvascular invasion, *PA* prealbumin, *PLT* blood platelets, *PT* prothrombin time, *TBiL* total bilirubin, *WBC* white blood cells

### Survival analysis

In univariable analyses, CK19 expression, presence of MVI, BCLC stage B, HBsAg positive, HBV-DNA ≥ 5 × 10^2^ IU/ml, large tumor diameter, tumor number ≥ 2, AFP ≥400 ng/ml, high N%, low L% , low Alb, low PA, high GGT, and high ALP were significantly associated with worse DFS after radical resection. In addition, CK19 expression, presence of MVI, HBV-DNA ≥ 5 × 10^2^ IU/ml, larger tumor diameter, incomplete envelope, presence of ascites, low PA, high GGT, and high ALP were significantly associated with worse OS (Tables 2 and 3).

**Table 2:** Univariable and multivariable analysis to identify predictors of disease-free survival of HCC patients after radical resection

Variable	Univariable				Multivariable			
	HR	95% CI		P	HR	95% CI		P
		Lower	Upper			Lower	Upper	
Age (years)	0.992	0.978	1.006	0.271				
Sex (male/female)	0.756	0.487	1.174	0.231				
BCLC stage (0/A/B)	1.637	1.224	2.189	0.001	1.018	0.609	1.703	0.994
HBsAg (negative/positive)	2.266	1.259	4.078	0.006	1.984	1.020	3.861	0.044
HBV-DNA (< 5 × 10^2^IU/ml/≧5 × 10^2^IU/ml)	1.656	1.179	2.325	0.004	1.398	0.945	2.068	0.094
Anti-HCV (negative/positive)	1.326	0.544	3.233	0.535				
Tumor diameter (cm)	1.115	1.068	1.164	< 0.001	1.060	1.007	1.116	0.026
Tumor number (1/≥2)	2.041	1.482	2.809	< 0.001	1.685	0.974	2.918	0.062
Tumor envelope (complete/incomplete)	1.067	0.721	1.580	0.746				
Ascites (negative/positive)	1.289	0.808	2.056	0.287				
AFP (<400 ng/ml/≥400 ng/ml)	1.450	1.071	1.963	0.016	1.207	0.877	1.660	0.249
WBC (*10^9^/L)	1.049	0.972	1.131	0.219				
N% (%)	1.020	1.004	1.037	0.014	1.009	0.976	1.044	0.583
L% (%)	0.975	0.958	0.993	0.008	0.997	0.960	1.035	0.869
HB (g/L)	1.001	0.992	1.010	0.853				
PLT (*10^9^/L)	1.000	0.998	1.002	0.909				
FPG (mmol/L)	1.081	0.985	1.185	0.099				
PT (s)	0.902	0.789	1.030	0.128				
INR	0.619	0.170	2.251	0.467				
TBiL (μmol/L)	0.982	0.958	1.007	0.150				
Alb (g/L)	0.964	0.931	0.997	0.033	0.988	0.948	1.030	0.584
PA (mg/L)	0.996	0.994	0.998	0.001	0.988	0.995	1.000	0.081
ALT (U/L)	0.998	0.994	1.003	0.485				
AST (U/L)	1.000	0.996	1.004	0.954				
GGT (U/L)	1.001	1.000	1.002	0.042	1.001	0.999	1.002	0.548
ALP (U/L)	1.004	1.002	1.006	0.001	1.002	0.999	1.005	0.146
CK19 (negative/positive)	1.437	1.021	2.022	0.038	1.604	1.100	2.337	0.014
MVI (negative/positive)	1.518	1.119	2.057	0.007	1.365	0.987	1.887	0.060

*Abbreviations*: *AFP* alpha fetoprotein, *Alb* albumin, *ALP* alkaline phosphatase, *ALT* alanine aminotransferase, *anti-HCV* antibodies against hepatitis C virus, *AST* aspartate aminotransferase; *BCLC* Barcelona Clinic liver cancer, *CK19* cytokeratin 19, *FPG* fasting plasma glucose, *GGT* γ-glutamyl transpeptidase, *HB* hemoglobin, *HBsAg* hepatitis B surface antigen, *INR* international normalized ratio, *L%* lymphocyte percentage, *N%* neutrophil percentage, *MVI* microvascular invasion; *PA* prealbumin, *PLT* blood platelets, *PT* prothrombin time, *TBiL* total bilirubin, *WBC* white blood cells

**Table 3:** Univariable and multivariable analysis to identify predictors of overall survival of HCC patients after radical resection

Variable	Univariable				Multivariable			
	HR	95% CI		P	HR	95% CI		P
		Lower	Upper			Lower	Upper	
Age (years)	0.993	0.974	1.012	0.449				
Sex (male/female)	0.716	0.399	1.286	0.264				
BCLC stage (0/A/B)	1.484	1.009	2.182	0.045				
HBsAg (negative/positive)	1.546	0.775	3.084	0.216				
HBV-DNA (< 5 × 10^2^IU/ml/≧5 × 10^2^IU/ml)	1.855	1.151	2.988	0.011	1.791	1.093	2.933	0.021
Anti-HCV (negative/positive)	0.455	0.063	3.284	0.435				
Tumor diameter (cm)	1.116	1.066	1.169	< 0.001	1.073	1.018	1.131	0.009
Tumor number (1/≥2)	1.415	0.913	2.193	0.121				
Tumor envelope (complete/incomplete)	2.281	1.459	3.569	< 0.001	2.169	1.368	3.440	0.001
Ascites (negative/positive)	1.862	1.055	3.286	0.032	1.659	0.922	2.985	0.091
AFP (<400 ng/ml/≥400 ng/ml)	1.237	0.824	1.857	0.305				
WBC (*10^9^/L)	1.006	0.909	1.112	0.913				
N% (%)	1.018	0.998	1.039	0.082				
L% (%)	0.977	0.955	1.001	0.056				
HB (g/L)	0.998	0.987	1.010	0.771				
PLT (*10^9^/L)	1.000	0.997	1.003	0.996				
FPG (mmol/L)	1.017	0.915	1.130	0.756				
PT (s)	1.041	0.876	1.237	0.651				
INR	1.658	0.310	8.872	0.554				
TBiL (μmol/L)	0.989	0.959	1.021	0.505				
Alb (g/L)	0.974	0.930	1.020	0.268				
PA (mg/L)	0.996	0.994	0.999	0.018	0.998	0.995	1.001	0.268
ALT (U/L)	0.997	0.989	1.004	0.396				
AST (U/L)	1.001	0.996	1.006	0.603				
GGT (U/L)	1.002	1.001	1.003	0.001	1.002	1.000	1.003	0.024
ALP (U/L)	1.006	1.003	1.008	< 0.001	1.004	1.001	1.008	0.026
CK19 (negative/positive)	1.641	1.060	2.540	0.026	1.471	0.936	2.313	0.094
MVI (negative/positive)	2.132	1.409	3.225	< 0.001	1.808	1.171	2.787	0.007

*Abbreviations*: *AFP* alpha fetoprotein, *Alb* albumin, *ALP* alkaline phosphatase, *ALT* alanine aminotransferase, *anti-HCV* antibodies against hepatitis C virus, *AST* aspartate aminotransferase, *BCLC* Barcelona Clinic liver cancer, *CK19* cytokeratin 19; *FPG* fasting plasma glucose, *GGT* γ-glutamyl transpeptidase, *HB* hemoglobin, *HBsAg* hepatitis B surface antigen, *INR* international normalized ratio, *L%* lymphocyte percentage, *N%* neutrophil percentage, *MVI* microvascular invasion, *PA* prealbumin, *PLT* blood platelets, *PT* prothrombin time, *TBiL* total bilirubin, *WBC* white blood cells

In multivariable analysis, CK19 expression, HBsAg positive and larger tumor diameter, but not presence of MVI, were independent predictors of DFS (Table 2). Presence of MVI, HBV-DNA ≥ 5 × 10^2^ IU/ml, larger tumor diameter, incomplete envelope, high GGT and high ALP, but not CK19 expression, were independent predictors of OS (Table 3).

**Fig. 2:**
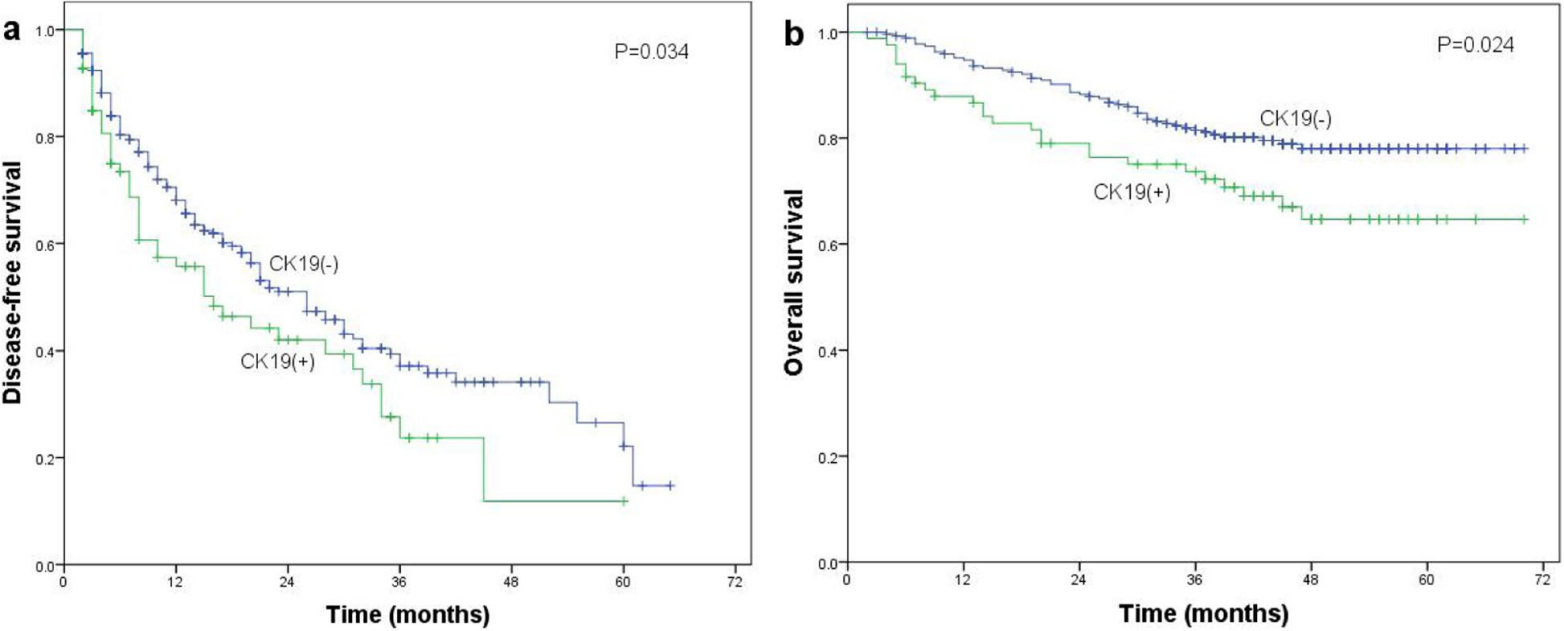
Association of CK19 expression with **a** disease-free survival or **b** overall survival of HCC patients after radical resection. Differences in the Kaplan–Meier curves were assessed for significance using the log-rank test

**Fig. 3:**
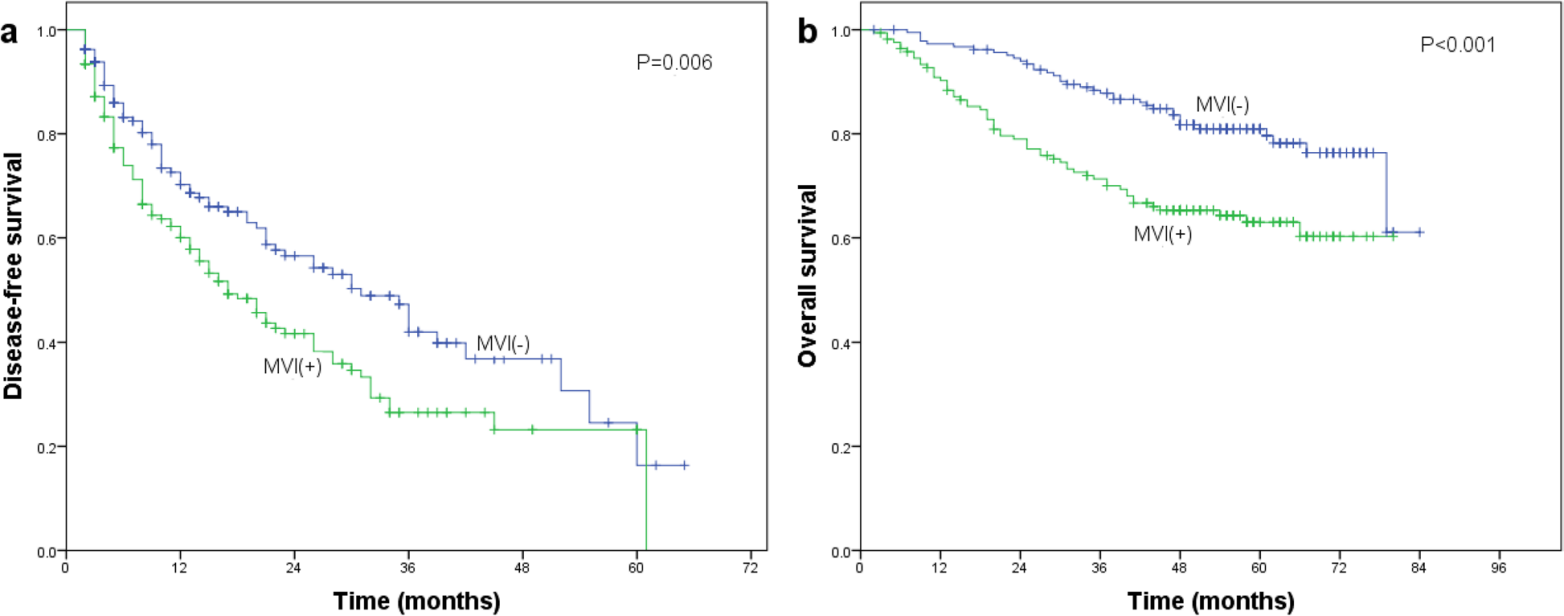
Association of MVI with **a** disease-free survival or **b** overall survival of HCC patients after radical resection. Differences in the Kaplan–Meier curves were assessed for significance using the log-rank test

On its own, CK19 expression was associated with significantly lower DFS (Fig. 2a) and OS (Fig. 2b) after radical resection. The same was observed for MVI on its own (Fig. 3a, b).

**Fig. 4:**
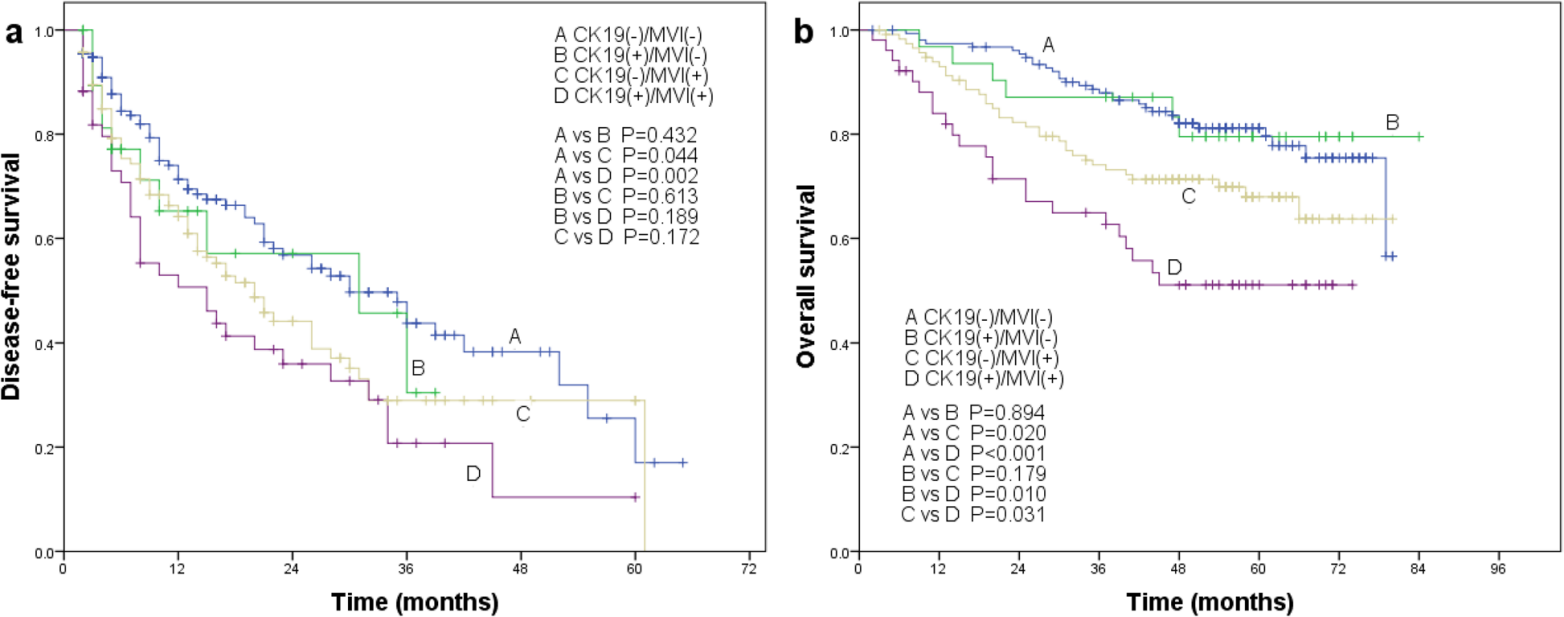
Association of the combination of CK19 expression and MVI with **a** disease-free survival and **b** overall survival in HCC patients after radical resection. Differences in the Kaplan–Meier curves were assessed for significance using the log-rank test

The combination of the two markers also showed a significant association with worse survival. DFS rate was significantly lower for CK19(+)/MVI(+) patients than for CK19(−)/MVI(−) patients, and it was significantly lower for CK19(−)/MVI(+) patients than for CK19(−)/MVI(−) patients (Fig. 4a). No other pairs of the four groups differed significantly in DFS rate. Similarly, OS rate was significantly lower for CK19(+)/MVI(+) patients than for the other three groups, while it was significantly higher for CK19(−)/MVI(−) patients than for CK19(−)/MVI(+) or CK19(+)/MVI(+) patients (Fig. 4b).

## Discussion

Here, we provide evidence that combining CK19 expression and MVI, each of which on its own predicts poor prognosis in HCC patients, may better predict the survival of such patients after potentially curative hepatic resection. DFS was significantly worse for CK19(+)/MVI(+) patients than for CK19(−)/MVI(−) patients, and OS was significantly worse for CK19(+)/MVI(+) patients than for patients who were negative for one or both of these markers. Our results are consistent with other studies showing that combinations of biomarkers often predict prognosis better than the individual biomarkers on their own [16, 18]. Our findings may help personalize the management of HCC patients, improving their long-term outcomes.

CK19, a marker of biliary/progenitor cells, is expressed in 10-20% of patients with HCC [13, 14, 19, 26], and the prevalence in our cohort was 23%. Our CK19(+) patients were younger and had higher levels of AFP and more MVI than CK19(−) patients. CK19(+) HCC seems to be more aggressive than CK19(−) disease and to involve higher risk of relapse and worse postoperative prognosis, which we observed in the present cohort. This is consistent with previous studies [27, 28].

CK19 expression may be associated with worse prognosis because tumor cells expressing that protein show stem cell characteristics of self-renewal [21, 29, 30]. In HBV-related HCC, cadherin 17 (CDH17) is significantly correlated with CK19 in primary tumor tissue. Epidermal growth factor can induce the expression of both CK19 and CDH17, and CDH17 in turn can enhance the expression of CK19 in HCC. Thus, expression of CDH17 may be associated with the early recurrence and poor prognosis of CK19(+) HCC [18]. One study of 237 cases of HCC found that CK19 was significantly associated with expression of EMT-related proteins, leading the investigators to propose that CK19 up-regulates EMT-related genes to make the cancer more invasive [19]. Other studies have suggested that the invasiveness of CK19(+) HCC may be related to expression of genes related to invasion and metastasis, to genes characteristic of biliary or hepatic progenitor cells and to microRNA 200 family members [13].

MVI is a mark of aggressive biological behavior and is associated with worse DFS and OS after liver resection or transplantation [6]. Patients with recurrent liver cancer also obtained similar results [31]. Some scholars even believe that the impact of MVI on prognosis is the same as that of gross vascular invasion confined to a segmental/sectional branch [32]. Our MVI(+) patients had larger tumors, lower prevalence of an intact tumor envelope, higher APF levels and worse DFS and OS than MVI(−) patients, consistent with previous results [7, 33]. Studies had shown that elderly patients with HCC were more prone to vascular invasion, which was not consistent with our findings [34]. Gross vascular invasion is usually a consequence of MVI progression. The poor prognosis of HCC with gross vascular invasion has been clarified, but it has a relatively large impact on the prognosis, which is not conducive to the accurate classification of the prognosis [35]. Therefore, we advocate combining CK19 and MVI to analyze the prognosis.

In fact, CK19(+) HCC seems to be associated with MVI. In our study, the prevalence of MVI was significantly higher among CK19(+) patients than CK19(−) patients (62.2% vs 43%). In a previous study, 73.5% of CK19(+) HCC patients had MVI, significantly more than the 56.8% of CK19(−) HCC patients with MVI [19]. In another study, MVI was more frequent among HCC patients expressing CK19, both in the surgical specimen cohort (100.0% vs 52.0%) and needle biopsy specimen cohort (66.7% vs 21.7%) [13]. Univariable analysis found that CK19 and MVI were significantly associated with worse DFS and OS. However, in multivariable analysis, MVI did not independently predict DFS, while CK19 did not independently predict OS. Thus, using CK19 or MVI on their own to predict prognosis has limitations, arguing for using the combination of the two.

Using the combination of CK19 and MVI, we found that OS was significantly lower for CK19(+)/MVI(+) patients than for CK19(+)/MVI(−) and CK19(−)/MVI(+) patients, suggesting an additive effect. In contrast, we did not find evidence that CK19 and MVI exert an additive effect on DFS, since the DFS rate of CK19(+)/MVI(+) patients did not differ significantly from those of CK19(+)/MVI(−) or CK19(−)/MVI(+) patients. This may reflect that CK19(+)/MVI(+) HCC patients progress faster after tumor recurrence, leading to shorter survival. Therefore, research on recurrent HCC is key to improving OS of patients.

We also found that the deleterious effects of CK19 on prognosis did not fully manifest unless tumor cells had invaded microvessels. Once they invade, tumor cells expressing CK19 may behave as stem cells and migrate efficiently, eventually leading to a decline in survival. This may explain why the prognosis of HCC patients with CK19(+) and MVI(+) is worse than the prognosis of the other three groups.

The 5-year OS rate in our cohort was 72.5%, even higher than the 62.9% reported in another study that included patients from 2000 to 2017 [36]. This may be due to recent improvements in comprehensive treatment, as well as to selection bias that favored higher survival rates. Therefore, our results need to be verified in a larger, more diverse sample from multiple centers. In addition, data on CK19 and MVI in our study was provided by post-resectional histopathology, which had certain limitations in preoperative risk stratification assessment. However, there are abundant and reliable methods for preoperative prediction of CK19 and MVI, which can make up for this limitation to a certain extent [37–40].

Pathological features can help predict the prognosis of liver cancer. This study may have higher predictive power if we add more pathological information such as fibrolamellar HCC [41]. At the same time, how to improve the accuracy of CK19 and the detection rate of MVI in pathology is worth exploring [42].

In conclusion, our study suggests that CK19 expression and presence of MVI predict poor prognosis after radical resection of HCC, and the two markers jointly contribute to poor OS. Thus, combining CK19 and MVI may predict post-resection prognosis better than either factor on its own.

## Data Availability

The datasets used and/or analyzed during the current study are available from the corresponding author on reasonable request.
